# Acknowledgement to Reviewers of European Journal of Investigation in Health, Psychology and Education in 2019

**DOI:** 10.3390/ejihpe10010030

**Published:** 2020-01-16

**Authors:** EJIHPE Editorial Office

**Affiliations:** MDPI, St. Alban-Anlage 66, 4052 Basel, Switzerland

The editorial team greatly appreciates the reviewers who have dedicated their considerable time and expertise to the journal’s rigorous editorial process over the past 12 months, regardless of whether the papers are finally published or not. In 2019, a total of 26 papers were published in the journal, with a median time to first decision of 17.5 days and a median time from submission to publication of 43 days. The editors would like to express their sincere gratitude to the following reviewers for their generous contribution in 2019:
Abbas, AzharMartin, Stephanie L.Al-Lami, FarisMcCamley, JohnAllred, SarahMcClinchy, JaneAndreou, EleniMcFadden, SandraAntonopoulou, ViviMcMillan, JaniceArribas Marín, Juan ManuelMéndez Mateo, InmaculadaBaez, YolandaMira, José JoaquínBarbosa, David PerezMizuno, KohBarcham, RichardMugabi, Ivan K.Bolboacă, Sorana D.Mukherjee, SoumyadeepBungum, TimothyNa, In SeopBurnett, HarveyNapal, MaríaBurr, BrandonNatafgi, NabilC. de la Torre-Montero, JulioNg, Qin XiangCebrián, GiselaNguyen, Khai TheCeccatto, Vânia MarilandeNiakšu, OlegasChambers IV, EdgarNicolaou, ConstantinosChoate, Peter W.Nowacki, MarekCook, Amanda C.Núñez-Cacho Utrilla, PedroCori, JenniferOlushola, JoyceCupani, MarcosOuellette, Rachel R.Dibaba, DanielPecoraro, FabrizioDidham, RobertPérez-Lopez, AlbertoDoukakis, SpyrosPodovšovnik, EvaDrigas, Athanasios S.Potluka, OtoDziuba, SzymonPower, Thomas G.Egea Zerolo, BlancaPozo Rico, TeresaEiroa-Orosa, Francisco JoséRaheem, BamideleEl Ghoch, MarwanReid, EvaElmi, ChiaraReimers, Anne KerstinEngelhard, MatthewReiter, E. MirandaFang, DiRogge, Ronald D.Fernandez Salinero, SamuelRudnick, AbrahamField-Richards, SarahRuhle, SaschaFusheini, AdamRutkauskaite, RenataGardner, LaurenRyabov, IgorGębski, JerzySalessi, SolanaGeorgalos, KonstantinosSalmerón-Manzano, EstherGeorgiadou, Elissavet GinaSan Pedro Veledo, María BelénGerke, OkeSantoro, Paolo EmilioGómez-de-Terreros-Guardiola, MontserratSchindler, LenaGoto, KenichiSeeman, Mary V.Guzik, AgnieszkaSerra, LídiaGyörkös, ChristinaShah, AjitHermanussen, MichelSiu, FionaHoudmont, JonathanSoares Teles, ArielHughes, VickieSperlì, GiancarloHurstel, AlexandreStará, JanaIrsara, MartinaSteen, S. (Steffie) Van DerItatani, TomoyaȘtefan, Simona CătălinaJones, Lise ØenStewart, ThomasKantono, KevinStrickland, PaulKaratsidis, AngelosTararova, OlgaKatona, JozsefTokarz, AleksandraKatsanos, Aristeidis H.Toscano, FerdinandoKayser, LarsToselli, StefaniaKelly, KimberlyTyburski, ErnestKim, Byung-JikUlibarri Ochoa, AinhoaKira, GeoffVan Hoof, ChrisKirchner, KathrinVarelas, VassileiosLauridsen, Jørgen TrankjærVila-Vázquez, GuadalupeLerma, ClaudiaVillafaina, SantosLerman, DorotheaVolenzo, Tom ElijahLewko, JolantaVoroshilova, AnzhelikaLi, WeijuanWanchai, AusaneeLiu, ChangqiWeigelt, OliverLlorente-Alonso, MartaWenos, JeanneLochbaum, MarcWilson, JasonLuis Ubago, JoseWren, DouglasM. Silva, FernandaYeh, Jun-JunMagnavita, NicolaZakariya, Yusuf F.Marcolin, FedericaZhao, ShuoMarker, Sandra


